# A Series of Oleanolic Acid Derivatives as Anti-Hepatitis B Virus Agents: Design, Synthesis, and *in Vitro* and *in Vivo* Biological Evaluation

**DOI:** 10.3390/molecules21040402

**Published:** 2016-03-24

**Authors:** Wenqiang Yan, Chenze Zhang, Bi Li, Xin Xu, Miao Liang, Shun Gu, Fuhao Chu, Bing Xu, Jian Ren, Penglong Wang, Haimin Lei

**Affiliations:** 1 School of Chinese Pharmacy, Beijing University of Chinese Medicine, Beijing 100102, China; ywq3226925@163.com (W.Y.); zcz920418@163.com (C.Z.); libimegan@163.com (B.L.); xu.xin@vip.126.com (X.X.); mimi1111202@sina.com (M.L.); chufhao@163.com (F.C.); weichenxubing@126.com (B.X.); renjian2008333@163.com (J.R.); 2 Department of Pharmacology, Xuanwu Hospital of Capital Medical University, Key Laboratory for Neurodegenerative Diseases of Ministry of Education, Beijing 100053, China; gszy2003@163.com

**Keywords:** oleanolic-acid derivatives, epoxy ring, eosin Y, anti-hepatitis B virus, virus recrudescence

## Abstract

A series of oleanolic acid derivatives were synthesized by diverse reactions, including the introduction of conjugated alkadiene and epoxy ring moieties formed by means of photosensitized oxidation. Eosin Y was used as photosensitizer during this process. Next the cytotoxicity of the products was evaluated on HepG2.2.15 cells to determine the appropriate treatment concentration for the subsequent experiments. Most of the **OA** derivatives exhibited anti-HBV antigens secretion activity in HepG2.2.15 cells. Among the tested compounds, **OA-4** (3.13 µg/mL) showed significant activity against the secretion of HBsAg, HBeAg, and HBV DNA replication with inhibitory ratios of 90.52% ± 1.78%, 31.55% ± 3.65%, and 94.57% ± 3.11% after 6 days, respectively. Besides, **OA-4** was further investigated in a duck model with DHBV infection. When **OA-4** was administered at a dosage of 500 mg/kg, the results revealed a significant inhibitory effects of DHBV at 19.94% ± 2.87%, 28.80% ± 3.62% and 29.25% ± 2.65% at days 5, 10, and 3 after the cessation of **OA-4** treatment, respectively. It’s worth noting that **OA-4** is superior to lamivudine in the inhibition of rebound of viral replication rate. The structure–activity relationships of **OA** derivatives had been preliminary discussed, which should be useful to explore further novel anti-HBV agents.

## 1. Introduction

Hepatitis B virus (HBV) infection is a serious worldwide public health problem which often leads to acute and chronic consequences in humans such as cirrhosis and hepatocellular carcinoma (HCC) [1,2,3]. Currently, there are several kinds of therapy which serve as effective therapeutic methods for treating HBV [4,5] such as immunomodulators, interferon-α and nucleoside analogues (NAs) whose advantages of relatively infrequent side effects and easy oral administration make NAs including lamivudine, telbivudine and entecavir (nucleoside analogues), and adefovir dipivoxil and tenofovir disoproxil fumarate (nucleotide analogues) popular treatment options [6]. However, millions of the HBV patients will eventually succumb to the infection sequence due to high recurrence, drug resistance and side effects, including influenza-like illness, myalgia, reduction of neutrophilic granulocytes and so on [7,8]. Therefore, the continued development of new antimicrobial agents with different antiviral targets and inhibition of viral load rebound is essential.

**Figure 1:**
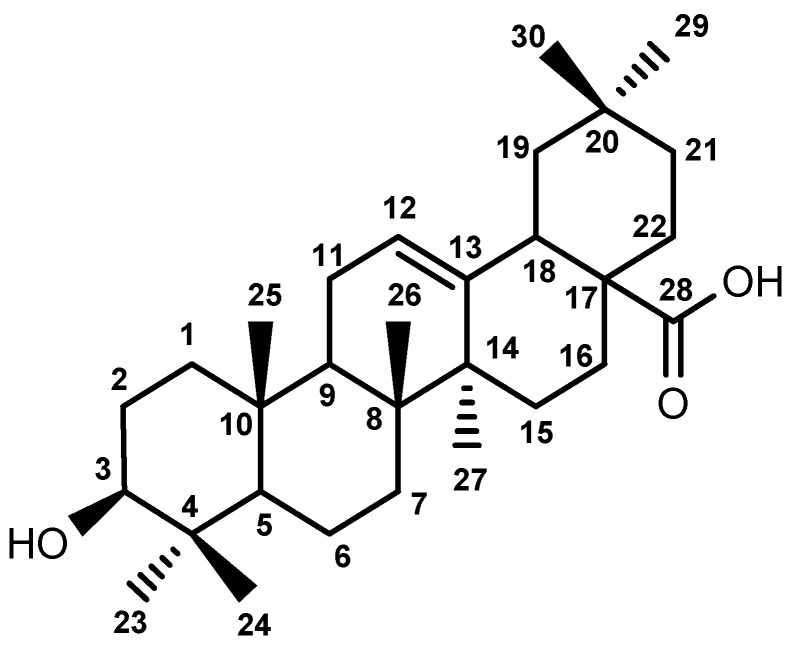
Structure of oleanolic acid.

Based on the clinical efficacy of traditional Chinese medicines, attempts to discover new lead compounds has already drawn considerable attention [9,10,11]. *Ligustrum lucidum* traditionally served as a natural liver-protection drug used for replenishing the liver and prolonging life [12,13]. Oleanolic acid (OA, Figure 1), obtained from *Ligustrum lucidum*, has been widely used in China for treating hepatopathy [14]. However, due to its natural product properties there still exist some issues that limit the clinical application of OA, such as poor hydrophilicity, poor bioavailability, *etc*. Therefore, more and more efforts have been spent on chemical modification and structure transformation of OA [15,16,17,18]. Some studies have provided reliable evidence suggesting that the introduction of 3-carbonyl and conjugated alkadiene groups results in more powerful anticancer activity [19,20]. Meanwhile, epoxy structures could enhance its antiviral activity. To facilitate the synthesis of this functional group, in the present work, we found a new method to obtain epoxy derivatives.

According to the above findings, in our continuing research for finding more potential HBV inhibitors, six compounds were synthesized. In this paper, we reported the synthesis, HBV inhibitory properties of these **OA** derivatives. Their structures were identified by high resolution-electrospray ionization (HR-ESI)-MS, one-dimensional (1D)- and two-dimensional (2D)-NMR assays.

## 2. Results

### 2.1. Chemistry

**Scheme 1:**
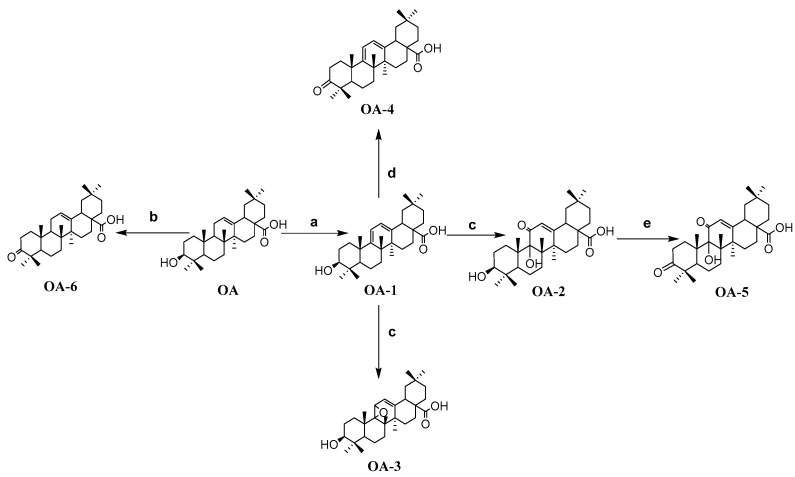
Synthesis route to **OA-n** derivatives. *Reagents and Conditions:* (**a**) NBS, CCl_4_, light, reflux, 4 h; (**b**) chromic acid solution, acetone, 0 °C, 1 h; (**c**) Eosin Y, dichloromethane, light, 10 h; (**d**) chromic acid solution, acetone, 0 °C, 1 h; (**e**) chromic acid solution, acetone, 0 °C, 1 h.

As shown in Scheme 1, **OA-1** was synthesized by the treatment of anhydrous **OA** with *N*-bromosuccinimide (NBS). **OA-1** was dissolved in dry DCM then Eosin Y is not an enzyme was added. At the same time, the mixture was kept illuminated for 10 h to give **OA-3** and **OA-2**, whose structures were further determined by HMQC and ^1^H-^1^H COSY 2D-NMR spectra. Combined with the significant HMQC, and ^1^H-^1^H COSY correlations, H-12 (δ 6.02, 1H, s) was connected with C-12 (δ 121.2), and H-12 (δ 6.02, 1H, s) was connected with H-18 (δ 1.52, 1H, m). Therefore, it is doubtless that a carbonyl was introduced at C-11. Moreover, OA-2 was dissolved in dry acetone then Jones’ reagent was added dropwise and **OA-5** was obtained. Similarly, **OA-4** and **OA-6** were obtained in the same way as **OA-5**, namely **OA-1** and **OA** were oxidized directly with Jones’ oxidation reagent, respectively. The structures of the compounds were elucidated by HRMS and NMR spectroscopy.

### 2.2. Biological Evaluation

#### 2.2.1. Cytotoxic Effect of Oleanolic Acid Derivatives on HepG2.2.15 Cells

**Figure 2:**
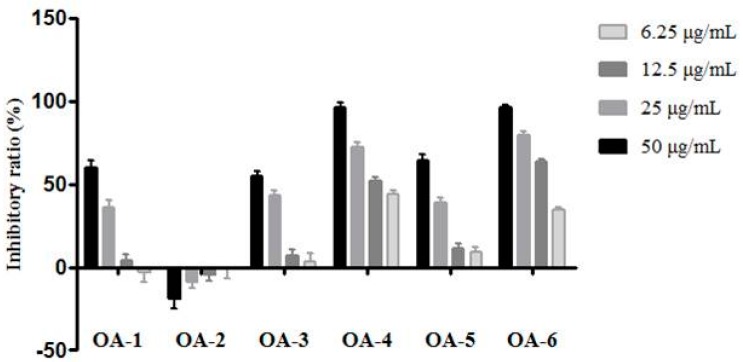
Cytotoxic effect of **OA-n** on HepG2.2.15 cell line. Data were expressed as inhibitory ratio ± SD based on three independent experiments.

The cytotoxicity of the oleanolic acid derivatives **OA-n** was evaluated by a MTS assay [21] on HepG2.2.15 cell lines. The inhibition ratios of the HepG2.2.15 cells exposed to different concentrations of **OA-n** are shown in Figure 2. The results are the mean of 50% cytotoxic concentration (TC_50_) and maximum non-toxic concentration (TC_0_) from the dose–response curves (Table 1).

These results were used to determine the appropriate **OA-n** treatment concentrations for the subsequent experiments.

#### 2.2.2. Activity against HBV Antigen Secretion in HepG2.2.15 Cells

**Figure 3:**
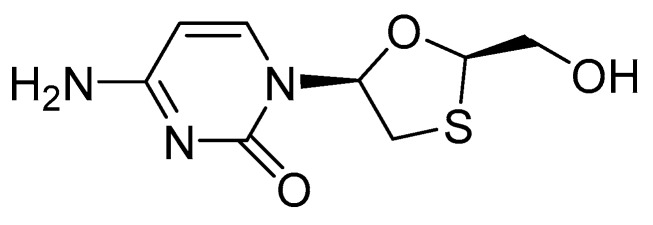
The structure of lamivudine (**3TC**).

**Figure 4:**
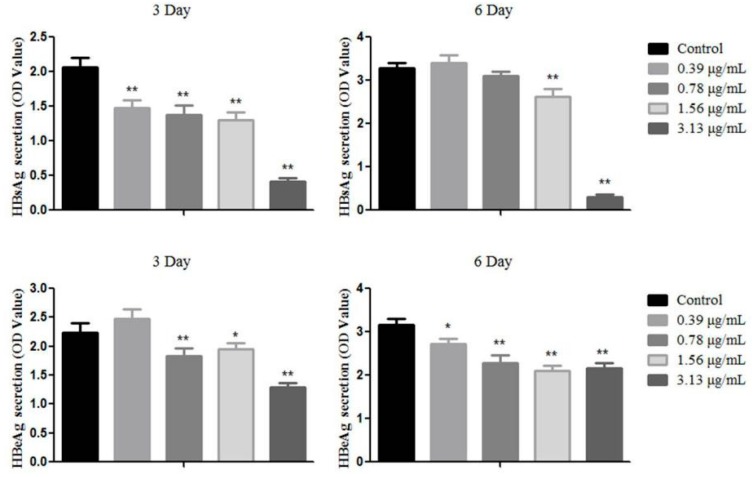
The activity against HBV antigen secretion of **OA-4** in HepG2.2.15 cells. Data are expressed as OD value ± SD based on three independent experiments. ** p < 0.01, * p < 0.05 compared with the control group.

The HepG2.2.15 cells were treated with **OA-n** at various concentrations to determine their inhibitory effects on the secretion of HBsAg and HBeAg after 3 or 6 days. Lamivudine (**3TC**, Figure 3) was used as positive control. The antigens in the culture supernatants were quantified using specific ELISA kits. The anti-HBV activity of each sample was shown by its inhibition of antigen secretion in HepG2.2.15 cells after treatment with the corresponding sample. The result demonstrated that **OA-3**, **OA-4**, **OA-5** can reduce HBsAg and HBeAg secretion at the 3rd or 6th day (Table 2). As shown in Figure 4, **OA-4** showed a significant inhibitory effect on HBsAg secretion, with the highest inhibition being 90.52% ± 1.78%. Like the HBeAg secretion, the secretion of HBeAg was significantly reduced, with the highest inhibition being 42.31% ± 3.53%.

#### 2.2.3. Effects of **OA-4** on HBV DNA Expression in HepG2.2.15 Cells

**Figure 5:**
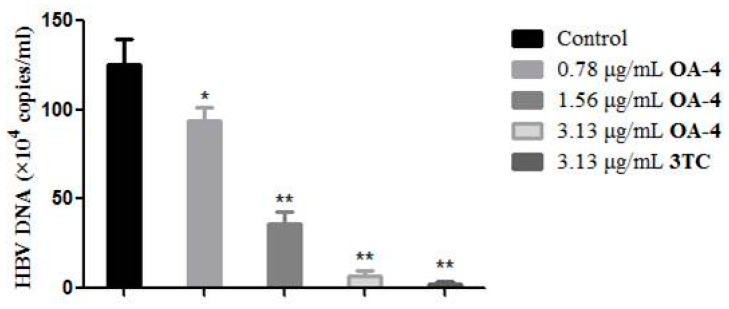
Inhibitory effect of **OA-4** and **3TC** on HBV DNA expression in HepG2.2.15 cells. HBV DNA levels were quantified by fluorescence quantitative PCR. Data are expressed as HBV DNA copies ± SD based on three independent experiments. ** p < 0.01, * p < 0.05 compared with the control group.

HBV is a DNA virus and HBV DNA testing is the most accurate indicator for measuring the contagiousness of hepatitis B. To further confirm the anti-HBV activity of **OA-4** in HepG2.2.15 cells, the effect of **OA-4** treatment on the level of HBV DNA was evaluated. As shown in Figure 5, **OA-4** showed significant inhibitory activity of HBV DNA expression after 6 days of treatment.

#### 2.2.4. *In Vivo* Anti-HBV Activity of **OA-4** in Ducks

**Figure 6:**
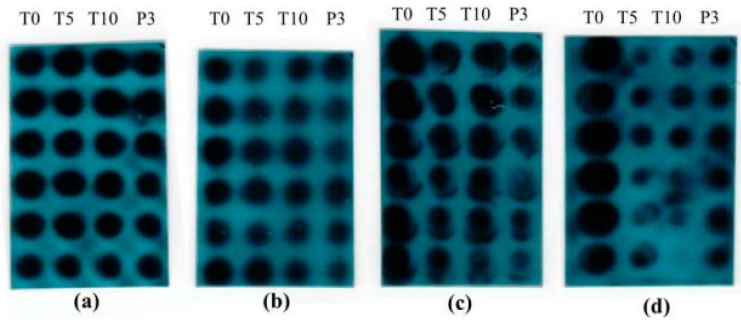
Dot blots of DHBV DNA in duck serum in the **OA-4** treatment study. (**a**) 125 mg/kg **OA-4** treatment; (**b**) 250 mg/kg **OA-4** treatment; (**c**) 500 mg/kg **OA-4** treatment; (**d**) 50 mg/kg **3TC** treatment.

**Figure 7:**
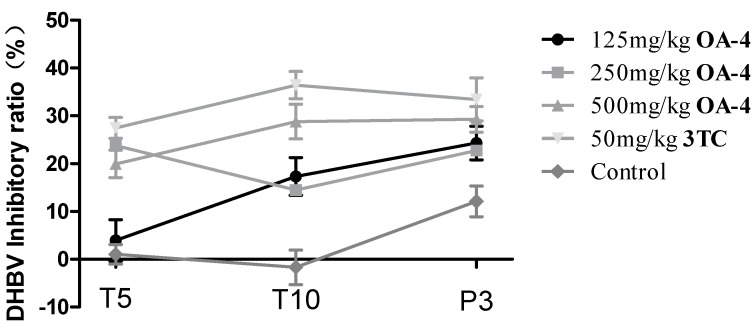
Inhibitory effect of **OA-4** on DHBV DNA expression in duck serum at days 5, 10, and 3 after the cessation of **OA-4** treatment. Data are expressed as DHBV DNA inhibitory ratio ± SD based on the independent experiments.

After treatment with **OA-4** and 3TC, serum DHBV DNA was analyzed by dot hybridization (Figure 6). The level of DHBV DNA of each treated group decreased significantly. As shown in Figure 7, the treatment with **OA-4** at the dose of 125, 250 and 500 mg/kg/day for 14 days exhibited a time and dose dependent inhibitory effect on DHBV DNA level. When **OA-4** at dosage of 500 mg/kg, densitometric quantitation of the dots revealed significant DHBV inhibitory effects of 19.94% ± 2.87%, 28.80% ± 3.62% and 29.25% ± 2.65% at days 5, 10, and 3 after the cessation of **OA-4** treatment (*n* = 6), respectively. A most noteworthy feature is the fact that the rebound of the DHBV DNA levels in **OA-4** treated ducks was less compared with the **3TC** treated group. No significant differences were observed in the DHBV DNA levels of any of the controls.

## 3. Discussion

In the synthesis process, a photosensitized oxidation reaction was utilized to prepare **OA-2** and **OA-3**, which was mediated by the sensitizer Eosin Y and was different from regular chemical oxidation. Eosin Y is a kind of organic dye [22], that has been used as the photosensitizer in recent years [23,24,25] due to its its favourable physical properties, good availability and low cost. The mechanism involved is probably that Eosin Y activates the oxydren in the air, and then the conjugated diene structure in **OA** was give to obtain the corresponding products. Besides, we analyzed the effect of the synthesized **OA** derivatives on the HepG2.2.15 cells. All six derivatives, except for **OA-2**, showed the higher cytotoxicity than **OA** in HepG2.2.15 cells. Among them, **OA-4**, **OA-5** and **OA-6** are the oxidation products of **OA-1**, **OA-2** and **OA**. What’s more, they all presented stronger biological activity than their precursor compounds. The result was accordance with the study of Huang [19], which suggested that 3-oxo oleanolic acid possesses stronger antitumor activity than **OA**. It was worth noting that **OA-2** showed no biological activity, whereas its oxidation product (**OA-5**) was shown to have anti-hepatitis B virus activity. This further proves that the introduction of a 3-carbonyl can enhance the effect of **OA** derivatives on hepatitis B virus. In addition, **OA-4** (derived from **OA-6**) and **OA-1** (derived from **OA**) possessed a conjugated alkene structure and showed stronger activities than **OA-6** and **OA**, which may suggest conjugated alkenes could be helpful to improve anti-hepatitis B virus activity. Among the derivatives, **OA-4**, possessing both the conjugated alkene structure and a 3-carbonyl, showed the most satisfactory activity which exceeded that of lamivudine. At the same time, **OA-4** was found to exert its effect of anti-hepatitis B virus in the inhibition of HBsAg, HBeAg and HBV DNA Expression. In the duck hepatitis B virus model **OA-4** hinders the rebound of viral replication rate. Meanwhile, oleanic acid, a pentacyclic triterpene, is probably different from the nucleoside analogs as far as the involved anti-HBV mechanism in concerned. 3TC, a nucleotide analog, displays a low genetic modification barrier for drug-resistant HBV and a high rate of clinical drug resistance. However, it was reported that oleanic acid and its derivatives serve as antiviral drugs, and could inhibit the entry and replication of the virus. The involved mechanism was probable that OA could act on the envelope protein, polymerase and protease [26,27,28]. Betulinic acid, whose structure is simliar to that of OA, could downregulate the expression of manganese superoxide dismutase and then inhibit HBV replication [29], so it is worth performing further studies on the effect of **OA** derivatives in suppressing the expression of the related enzymes. Based on the above, **OA-4** showed bright prospects.

## 4. Materials and Methods

### 4.1. General Information

Reactions were monitored by TLC using silica gel coated aluminum sheets (Qingdao Haiyang Chemical Co., Qingdao, China) and visualized in UV light (254 nm). ^1^H-NMR and ^13^C-NMR assays were recorded on an AVANCE 500 NMR spectrometer (Bruker, Fällanden, Switzerland) and chemical shifts are reported on the δ scale (ppm). Deuterated chloroform was used as the solvent (Beijing InnoChem Science and Technology Co., Ltd., Beijing, China). HRMS spectra were recorded on a high-resolution ESI mass spectrometer (RRLC 6320 Trap Iron MS, Agilent, Santa Clara, CA, USA). Samples were freeze-dried using a vacuum freeze dryer (Beijing Boyikang Co., Ltd., Beijing, China). Melting points were measured at a rate of 5 °C per minute using an X-5 micro melting point apparatus (Beijing Tech Instrument Co., Ltd., Beijing, China). Cellular morphologies were observed using an inverted fluorescence microscope (Olympus IX71, Tokyo, Japan). Silica-gel column chromatography was performed using 200–300 mesh silica gel. The yields were calculated based on the last step reaction. All solvents and chemicals used were analytical or high-performance liquid chromatography grade. HepG2.2.15 cells provided by the 302 Military Hospital of China were maintained in DMEM medium, supplemented with 10% fetal bovine serum (Hyclone, Aurora, ON, Canada) at 37 °C under humidified air with 5% CO_2_. HepG2.2.15 cells were derived from HepG2 cells and stably expresses HBV, has described in a previous study [30].

### 4.2. Chemistry

#### 4.2.1. Preparation of **OA-1**

OA (0.02 mol) and NBS (0.024 mol) were dissolved in dry tetrachloromethane (400 mL). The mixture was refluxed at 85 °C for 4 h with magnetic stirring, while at the same time, it was illuminated with incandescent bulb until the end of the experiment. The crude product was redissolved in an appropriate quantity of hot ethyl acetate and then filtered. The filtrate was cooled overnight in the refrigerator to obtain crude crystals which was then purified by recrystallization from ethyl acetate to yield the title compound *3β-hydroxyoleana-9(11),12-dien-28-oic acid* (**OA-1**). Yield: 58.5%, m.p.: 277.7–278.4 °C , white solid, ^1^H-NMR (CDCl_3_) (ppm): 0.83, 0.93, 0.97, 1.00, 1.05, 1.06, 1.20 (3H, each, s, 7 × CH_3_), 2.99 (m, 1H), 3.28 (m, 1H), 5.58 (d, 1H, *J* = 5.5 Hz), 5.62 (d, 1H, *J* = 5.5 Hz), 1.00–2.50 (21H, methines and methylene protons). ^13^C-NMR (CDCl_3_): 15.7, 18.2, 20.2, 20.4, 23.6, 23.6, 25.1, 27.0, 27.9, 28.3, 30.7, 32.2, 32.2, 32.9, 33.8, 37.1, 38.9, 38.9, 39.4, 40.7, 42.4, 45.9, 45.9, 51.2, 78.7, 115.6, 120.7, 144.9, 154.7, 183.5 (–C=O). HRMS (ESI) *m*/*z*: 455.32819 [M + H]^+^, calcd, for C_30_H_46_O_3_: 454.68444.

#### 4.2.2. Preparation of **OA-2** and **OA-3**

**OA-1** (0.01 mol) was dissolved in dry DCM (500 mL) then Eosin Y (200 mg) was added. The reaction system was kept illuminated for 10 h. When no unreacted starting material remained, the mixture reaction was washed with saturated sodium bicarbonate solution until it had a neutral pH. After drying the organic layer over anhydrous Na_2_SO_4_ and evaporating the solvent under vacuum, the crude product was purified by silica-gel column chromatography with petroleum ether–ethyl acetate (20:1) as eluent.

*3β,9-Dihydroxyoleana-12-en-28-oic acid* (**OA-2**). Yield: 40.2%, m.p.: 176.2–177.9 °C, white solid, ^1^H-NMR (CD_3_COCD_3_): 0.87, 0.98, 0.99, 1.02, 1.08, 1.28, 1.27 (3H, each, s, 7 × CH_3_), 3.01 (1H, m), 3.25 (1H, m), 6.02 (1H, s), 1.00–2.50 (22H, methine and methylene protons). ^13^C-NMR (CDCl_3_): 15.4, 17.1, 20.1, 22.5, 23.2, 24.0, 25.6, 27.3, 27.5, 27.6, 29.6, 31.3, 32.4, 33.7, 33.8, 36.3, 36.3, 39.2, 40.3, 41.6, 43.3, 44.0, 45.9, 50.1, 76.7, 87.5, 121.2, 177.6 (-C=O), 184.0 (-C=O), 191.7. HRMS (ESI) *m*/*z*: 487.28925 [M + H]^+^, calcd, for C_30_H_46_O_5_: 486.68324.

*3β-Hydroxyoleana-9,11-epoxy-28-oic acid* (**OA-3**). Yield: 20.3%, m.p.: 169.1–170.8 °C, white solid, ^1^H-NMR (CDCl_3_): 0.83, 0.96, 0.99, 1.03, 1.05, 1.20, 1.21(3H, each, s, 7 × CH_3_), 3.22 (1H, m), 4.33 (1H, d, *J* = 2.5 Hz), 5.38 (1H, d, *J* = 2.5 Hz), 1.00–2.50 (22H, methine and methylene protons). ^13^C-NMR (125 MHZ, CDCl_3_): 15.4, 17.5, 17.6, 20.7, 24.1, 24.5, 27.1, 27.2, 27.7, 27.8, 28.1, 28.2, 29.7, 32.9, 33.3, 32.3, 34.2, 36.9, 37.7, 44.02, 47.7, 51.41, 65.7, 72.3, 78.4, 78.5, 89.5, 118.0, 158.0, 179.4 (–C=O). HRMS (ESI) *m*/*z*: 471.34647 [M + H]^+^, calcd, for C_30_H_46_O_4_: 470.68384.

#### 4.2.3. General Synthesis of the Compounds **OA-4**, **OA-5** and **OA-6**

The corresponding reactant (**OA-1**, **OA-2** or **OA**, 1.5 mmol) was dissolved in dry acetone (50 mL). The reaction mixture was cooled to 0 °C by using an ice-water bath, and chromic acid (1.6 mmol) was added dropwise in 5 min. When there was no unreacted materials, the reaction solution was poured into a mixture of ice and water. After stirring, and filtration, the crude product was achieved. Then the crude product was washed with a sufficient amount of alcohol to afford the purified compound.

*3-Oxoursoleana-9(11),12-dien-28-oic acid* (**OA-4**). Yield: 27.5%, m.p.: 154.4–155.8 °C, white solid, ^1^H-NMR (CDCl_3_) (ppm): 0.93, 0.97, 1.03, 1.06, 1.08, 1.13, 1.26 (3H, each, s, 7 × CH_3_) 5.61 (d, 1H, *J* = 6.0 Hz), 5.67 (d, 1H, *J* = 5.5 Hz) 1.00–2.50 (21H, methine and methylene protons). ^13^C-NMR (CDCl_3_): 19.5, 20.0, 20.1, 21.3, 23.6, 23.6, 26.0, 26.0, 27.0, 30.7, 31.3, 32.1, 32.9, 33.7, 34.5, 37.7, 38.3, 39.5, 40.7, 42.6, 45.8, 45.9, 47.3, 51.8, 117.4, 120.7, 145.4, 152.7, 182.7 (-C=O), 217.1 (-C=O). HRMS (ESI) *m*/*z*: 453.34317 [M + H]^+^, calcd, for C_30_H_44_O_3_: 452.66856.

*3,11-Dioxoursaoleana-9-hydroxy-28-oic acid* (**OA-5**). Yield: 95%, m.p.: 177.3–179.1 °C, white solid, ^1^H-NMR (500 MHz, CDCl_3_) (ppm): 0.95, 0.95, 1.00, 1.10, 1.13, 1.37, 1.49 (3H, each, s, 7 × CH_3_), 6.02 (1H, s), 1.00–2.50 (22H, methine- and methylene- of OA-5). ^13^C-NMR (CDCl_3_): 18.3, 20.2, 21.4, 23.0, 23.8, 24.3, 25.8, 26.3, 29.9, 31.6, 33.1, 33.1, 33.1, 34.0, 34.0, 36.7, 37.0, 39.8, 41.7, 43.5, 44.0, 46.1, 47.6, 50.8, 87.8, 122.8, 178.5, 182.7 (-C=O), 192 (-C=O), 215.4 (-C=O). HRMS (ESI) *m*/*z*: 507.27603 [M + Na]^+^, calcd, for C_30_H_44_O_5_: 484.66786.

*3-Oxoursoleana-12-en-28-oic acid* (**OA-6**). Yield: 73.15%, m.p.: 211.8–212.7 °C, white solid, ^1^H-NMR (CDCl_3_) (ppm): 0.83, 0.93, 0.95, 1.01, 1.05, 1.07, 1.19 (s, 3H, each, 7 × CH_3_), 2.87 (m, 1H), 5.32 (brs, 1H), 1.00–2.50 (23H, methane and methylene protons). ^13^C-NMR (CDCl_3_): 15.3, 15.6, 16.8, 18.3, 23.2, 24.4, 24.7, 25.9, 27.3, 27.7, 28.1, 30.6, 32.3, 32.5, 32.9, 33.3, 37.6, 38.5, 38.8, 38.8, 41.5, 41.8, 45.7, 45.9, 47.6, 54.2, 122.5, 144.6, 177.3 (-C=O), 219 (-C=O). HRMS (ESI) *m*/*z*: 455.32831 [M + H]^+^, calcd, for C_30_H_46_O_3_: 454.68444.

### 4.3. Cytotoxicity Evaluation

Growing HepG2.2.15 cells were plated at a density of 1 × 10^5^ cells/mL and incubated in 96-well plates for 24 h (37 °C, 5% CO_2_). Then the cells were treated with different concentrations (6.25, 12.5, 25 or 50 μg/mL) of **OA-n** for 3 or 6 days. The cytotoxicity of oleanolic acid derivatives was evaluated by MTS assay. MTS solution was added to each well, and the plate was incubated for a further 3 h. The absorbance was read at 490 nm with a BIORAD 550 spectrophotometer (BioRad, Hercules, CA, USA). Wells containing no drugs were used as negative controls. Cell growth inhibitory ratio (%) was calculated using the following equation:

(1) Inhibition % = (1 − Sample group OD/Control group OD) × 100%

### 4.4. Assay of HBsAg and HBeAg Secretion

HepG2.2.15 cells were seeded in 96-well plates at a density of 1 × 10^4^ per well for the measurement of HBV antigens. After incubation with various concentrations of **OA-n**, or **3TC** for 3 or 6 days, the culture medium was collected, cell debris was removed, and the result was stored at −20 °C until analysis. The levels of Hepatitis B surface antigen (HBsAg) and Hepatitis B e antigen (HBeAg) in culture supernatants of HepG2.2.15 cells were determined using the enzyme-linked immunosorbent assay (ELISA) kit according to the manufacturer’s protocol (DaAn Gene Corp. of Sun Yat-sen University, Guangzhou, China). The optical density (OD) was measured at 450 nm using a BIORAD 550 spectrophotometer (BioRad). Inhibition ratio (%) was calculated based on Equation (1).

### 4.5. Quantification of HBV DNA in the Culture Medium

HepG2.2.15 cells were treated with compound **OA-4** and **3TC** for 3 or 6 days as described above. To isolate HBV DNA from the HepG2.2.15 cells, HBV DNA was extracted from culture supernatants and quantified by a HBV fluorescence quantitative PCR diagnostic kit (DaAn Gene Corp. of Sun Yat-sen University) according to the manufacturer’s instructions. In brief, a mixture of the cell culture supernatants or amplification standards and 50 μL of the reaction mixture containing 1.0 μM primer, 0.1 μM fluorescent probe, 200 μM DNA, 50 nkat Taq DNA polymerase, and 1× buffer were placed in a 0.2 mL reaction tube. The reaction tube was heated to 93 °C for 2 min pre-denaturation, then 93 °C for 45 s and 55 °C for 60 s, during the first 10 cycles, followed by 30 cycles at 93 °C for 30 s and 55 °C for 45 s. HBV DNA copies from HepG2.2.15 cells were calculated based on the Ct value and standard curves. HBV DNA inhibitory ratio (%) was calculated according to the following equation:

(2) Inhibition % = (1 − Sample group copy number/Control group copy number) × 100%

### 4.6. Experimental Inoculations of Ducklings

Each one-day-old Peking ducks were injected in the tibial vein with 0.2 mL of serum from ducks with positive DHBV DNA serology (7.5 × 10^7^ viral genome equivalent). The drug treatment experiment was carried out 7 days after ducks were infected with DHBV, and then the positive ducks were randomly divided into five groups. Compound **OA-4** was solubilized in isotonic saline solution and administered orally in a liquid diet. The drug groups were treated with three dosages of **OA-4** (125, 250, 500 mg/kg, twice daily). The isotonic saline liquid diet was also administered to the animals as negative control. **3TC** (50 mg/kg, twice daily) was used as positive control. Blood samples were taken at initiation of treatment (T0), the fifth day of treatment (T5), the tenth day of treatment (T10) and the third day (P3) of post-treatment follow-up. The serum was stored at −80 °C until analysis.

### 4.7. Dot Blot Analysis for DHBV DNA

DHBV DNA in the serum was measured by dot blot. Briefly, 40 μL of duckling serum was directly spotted on the nitrocellulose membrane (Amersham Biosciences Corp., Piscataway, NJ, USA) and DHBV DNA was detected with α-32P deoxycytidine labeled full-length DHBV genomic DNA. Incorporation of radioactivity was determined by Molecular Dynamics storage phosphor screen cassette (Amersham Biosciences Corp.). DHBV DNA was quantified by using the Gel-Pro analyzer4.0 software (Media Cybernetics Inc., Bethesda, MD, USA). DHBV DNA inhibitory ratio (%) was calculated in the following equation:

(3) Inhibition % = (1 − Sample group OD/Control group OD) × 100%

## 5. Conclusions

In summary, a series of novel oleanolic acid derivatives were synthesized using photosensitized oxidation reactions, which could be regarded as a new, low-cost, and environmentally friendly method to form epoxy rings. The target products were characterized by spectroscopic data. Their cytotoxicity was evaluated on HepG2.2.15 cells by the standard MTS assay and most of the derivatives displayed potent anti-HBV activity. Besides, all tesed compounds inhibited the secretion of HBsAg and **OA-3**, **OA-5**, **OA-4**, **OA-6** not only inhibited the secretion of HBsAg, but also decreased the secretion of HBeAg. In addition, **OA-4** exhibited significant inhibition of HBV DNA replication, superior to that of the reference drug. The experiments on duck hepatitis B virus (DHBV) indicated that **OA-4** has advantages over 3TC in the inhibition of viral replication rate rebound. Due to its good performance *in vivo* and vitro, **OA-4** may be potential novel anti-HBV drug candidate with a unique mechanism of action worthy of further investigation.

## Figures and Tables

**Table 1:** TC_50_ and TC_0_ values (µg/mL) of **OA-n** on HepG2.2.15 cell line.

Compound	TC_50_ (µg/mL)	TC_0_ (µg/mL)
**OA-1**	38.15	8.64
**OA-2**	>50	>50
**OA-3**	37.82	7.21
**OA-4**	8.64	1.04
**OA-5**	33.25	6.41
**OA-6**	10.33	2.18

**Table 2:** Inhibitory effect of **OA-n** on HBsAg and HBeAg secretion in HepG2.2.15 cells. Data are expressed as inhibitory ratio ± SD based on three independent experiments.

Compound	Concentration (μg/mL)	3 Day		6 Day	
		HBsAg (%)	HBeAg (%)	HBsAg (%)	HBeAg (%)
**OA-1**	6.25	5.03 ± 1.42	10.57 ± 3.72	0.42 ± 1.56	−10.01 ± 4.37
**OA-2**	6.25	−11.96 ± 3.21	−29.0 ± 0.46	−6.67 ± 2.40	−20.33 ± 7.53
**OA-3**	6.25	17.53 ± 3.09	1.15 ± 1.31	17.33 ± 4.74	3.56 ± 2.12
**OA-4**	1.56	36.48 ± 4.82	13.03 ± 3.17	20.21 ± 3.67	33.68 ± 4.03
**OA-5**	6.25	16.68 ± 4.64	6.78 ± 3.24	13.22 ± 2.83	8.54 ± 3.95
**OA-6**	1.56	−15.50 ± 5.80	−16.88 ± 5.96	3.76 ± 1.65	−30.06 ± 7.32
**3TC**	6.25	4.68 ± 2.73	6.31 ± 4.31	6.85 ± 2.58	−16.12 ± 4.55

## Abbreviations

The following abbreviations are used in this manuscript:

DCM	dichloromethane
NBS	*N*-bromosuccinimide
